# A Family With Discordant Germline BRCA1 and BRCA2 Variants: A BRCA1-Positive Mother and BRCA2-Positive Daughter With Possible Paternal Inheritance

**DOI:** 10.7759/cureus.116170

**Published:** 2026-09-13

**Authors:** Masahiro Kanamori, Hiroshi Adachi, Misa Kobayashi, Satoshi Hirakawa, Hiroharu Kobayashi

**Affiliations:** 1 Obstetrics and Gynecology, Seirei Hamamatsu General Hospital, Hamamatsu, JPN

**Keywords:** brca1, brca2, cascade testing, germline testing, hereditary cancer, paternal inheritance, primary peritoneal carcinoma

## Abstract

Hereditary breast and ovarian cancer (HBOC) is primarily caused by germline pathogenic variants in BRCA1 or BRCA2. Although a single pathogenic variant usually explains cancer predisposition within a family, independent pathogenic variants may coexist and complicate risk assessment and genetic counseling. We report a 43-year-old woman with right breast cancer in whom germline genetic testing identified a pathogenic BRCA2 variant, c.2808_2811del (p.Ala938ProfsTer21). Her mother developed primary peritoneal carcinoma at the age of 77. Tumor genomic testing identified a BRCA1 deletion of exons 1-12, which was subsequently confirmed as a germline variant. No pathogenic BRCA2 variant was detected in the mother. These discordant findings suggest that the daughter’s BRCA2 variant may have been inherited through the paternal lineage or may have arisen de novo. The father was deceased and unavailable for genetic testing, and the paternal family history was uninformative. This case demonstrates that multiple independent BRCA pathogenic variants may coexist within a single family. Restricting cascade testing to a single known familial variant may therefore result in incomplete hereditary cancer risk assessment. Comprehensive germline evaluation and individualized, pedigree-based genetic counseling should be considered when clinical findings are discordant with a known familial variant.

## Introduction

Hereditary breast and ovarian cancer (HBOC) syndrome is mainly caused by germline pathogenic variants in BRCA1 or BRCA2. BRCA1/2-associated HBOC is characterized by increased risks of female and male breast cancer, ovarian cancer, including fallopian tube and primary peritoneal cancers, and, particularly for BRCA2 carriers, pancreatic and prostate cancers [1]. Prospective cohort data have shown high cumulative breast cancer risks to age 80 years for both BRCA1 and BRCA2 carriers, with higher ovarian cancer risk in BRCA1 carriers than in BRCA2 carriers [2].

Current clinical practice increasingly emphasizes germline testing and family-based risk assessment. The American Society of Clinical Oncology (ASCO)-Society of Surgical Oncology (SSO) guideline recommends BRCA1/2 testing for all newly diagnosed breast cancer patients aged 65 years or younger and selected older patients based on clinical and family history or treatment implications [3]. The National Comprehensive Cancer Network (NCCN) guidelines similarly address genetic counseling, testing, and risk management for individuals with pathogenic or likely pathogenic variants associated with breast, ovarian, pancreatic, and prostate cancer risk [4].

In most HBOC families, a single familial pathogenic variant explains the inherited cancer predisposition. However, double heterozygosity and the co-occurrence of pathogenic variants across hereditary cancer genes have been reported, particularly with the increasing use of multigene panel testing [5-7]. Furthermore, cascade testing is often performed as targeted testing for a known familial variant, which is efficient but may not identify additional variants outside the targeted region [8]. Here, we report a family in which a mother carried a germline BRCA1 deletion of exons 1-12 and her daughter carried a distinct germline BRCA2 frameshift variant, raising the possibility of paternal inheritance of the daughter’s BRCA2 variant despite an uninformative paternal cancer history, while a de novo origin could not be excluded.

## Case presentation

A 43-year-old woman presented with right breast cancer in 2019. She underwent nipple-sparing mastectomy and sentinel lymph node biopsy. Pathological examination revealed invasive ductal carcinoma, pT1cN0, with luminal B features. The invasive diameter was 11 mm, with no sentinel lymph node metastasis. Immunohistochemistry showed estrogen receptor positivity, progesterone receptor positivity, HER2 equivocal by immunohistochemistry but negative by dual in situ hybridization (DISH), and a high proliferative index. She received adjuvant chemotherapy followed by tamoxifen.

In 2020, she developed a local recurrence and underwent resection followed by radiotherapy. The recurrent lesion was consistent with recurrent invasive ductal carcinoma. In March 2025, germline genetic testing using BRACAnalysis, which included sequencing and large rearrangement analysis of both BRCA1 and BRCA2, identified a pathogenic BRCA2 variant, c.2808_2811del (p.Ala938ProfsTer21). The laboratory report classified this variant as deleterious and clinically significant. The available clinical laboratory report did not specify whether orthogonal confirmation was performed or explicitly state the variant-classification framework used. No pathogenic BRCA1 variant was detected; specifically, the maternal BRCA1 deletion of exons 1-12 was not detected in the daughter. She was diagnosed with HBOC and subsequently underwent risk-reducing salpingo-oophorectomy. Pathological examination using the Sectioning and Extensively Examining the FIMbriated End (SEE-FIM) protocol showed no malignancy, including no serous tubal intraepithelial carcinoma.

Her mother was diagnosed with primary peritoneal carcinoma at the age of 77 in 2022. Ascitic fluid cytology showed adenocarcinoma. Cell block analysis demonstrated atypical papillary cell clusters consistent with carcinoma dissemination. Immunohistochemistry showed PAX8 positivity, partial estrogen receptor positivity, and TTF-1 negativity, supporting Müllerian origin. She was diagnosed with stage IIIC primary peritoneal carcinoma.

Tumor genomic testing using myChoice CDx (Myriad Genetic Laboratories, Inc., Salt Lake City, Utah, USA) in 2023 identified a BRCA1 deletion of exons 1-12. Because tumor testing alone does not establish germline inheritance, germline testing was later performed using BRACAnalysis. Blood was collected in June 2025, after the daughter’s germline testing, and the maternal germline BRCA1 deletion of exons 1-12 was confirmed. No pathogenic BRCA2 variant was detected in the mother.

The BRCA variants identified in the mother and daughter were distinct: a BRCA1 deletion of exons 1-12 in the mother and BRCA2 c.2808_2811del (p.Ala938ProfsTer21) in the daughter. No pathogenic BRCA2 variant was detected in the mother, indicating that the daughter’s BRCA2 variant was unlikely to have been maternally inherited. The father had died without BRCA testing. His age at death and detailed age information for paternal relatives were unavailable. No apparent paternal history of breast, ovarian, pancreatic, prostate, or other BRCA-associated cancer was identified. Consequently, the origin of the daughter’s BRCA2 variant could not be established. Paternal inheritance remains possible, but a de novo origin cannot be excluded.

The pedigree and germline BRCA findings of the family are shown in Figure 1.

**Figure 1:**
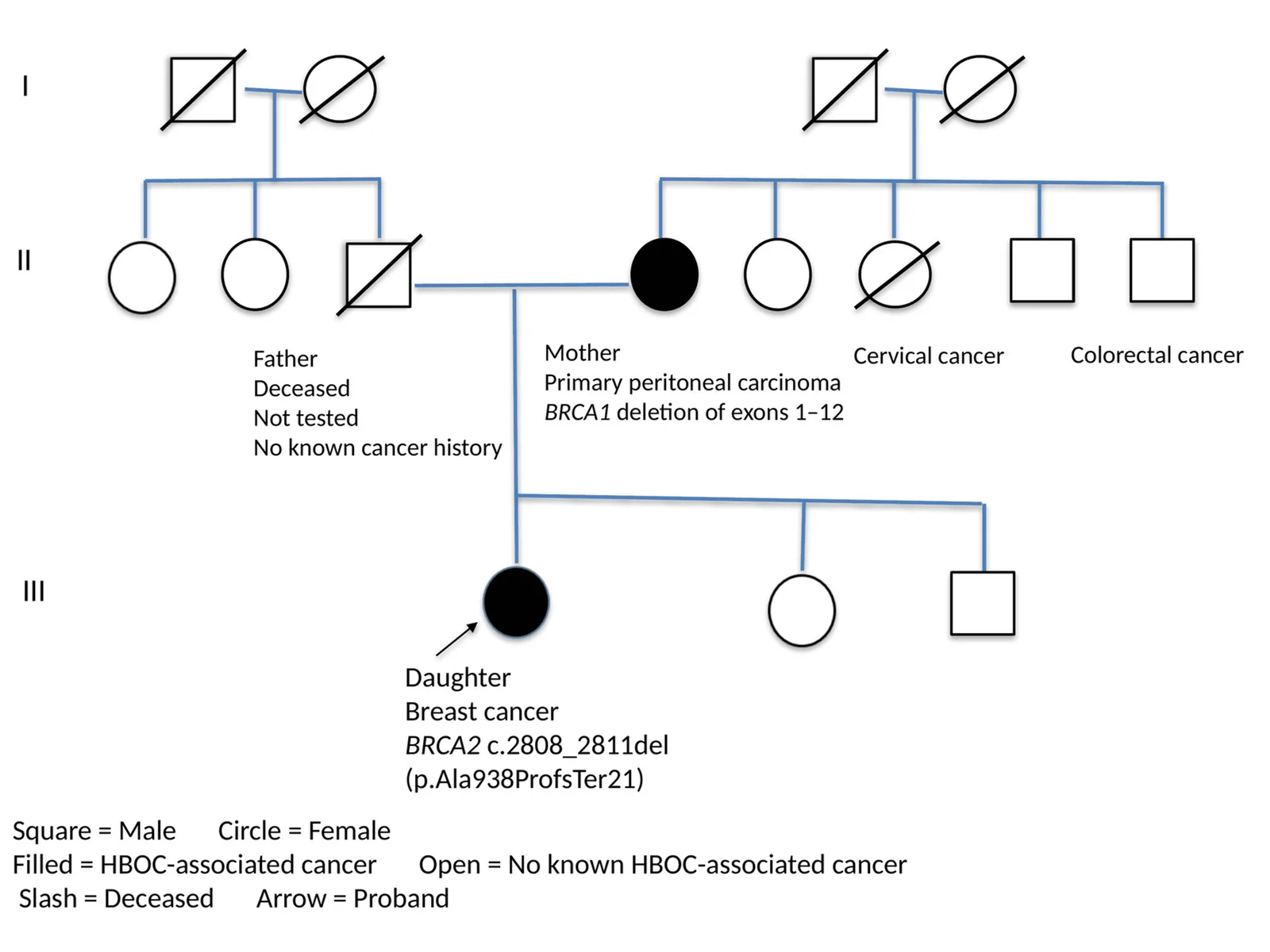
Pedigree of a family with discordant germline BRCA1 and BRCA2 pathogenic variants. Squares indicate males, circles indicate females, and diagonal slashes indicate deceased individuals. Filled symbols indicate individuals affected with an HBOC-associated cancer, whereas open symbols indicate individuals without a known HBOC-associated cancer. The arrow indicates the proband. The mother had primary peritoneal carcinoma and carried a germline BRCA1 deletion of exons 1-12. The daughter, who was the proband, had breast cancer and carried the germline BRCA2 pathogenic variant c.2808_2811del (p.Ala938ProfsTer21). The father was deceased, had not undergone genetic testing, and had no known history of BRCA-associated cancer; his age at death and detailed age information for paternal relatives were unavailable. The maternal BRCA1 deletion of exons 1-12 was not detected in the daughter, and the daughter’s BRCA2 variant was not detected in her mother. HBOC: hereditary breast and ovarian cancer

Representative histopathological and immunohistochemical findings from the daughter’s breast tumor and the mother’s ascitic-fluid cell block are shown in Figure 2.

**Figure 2:**
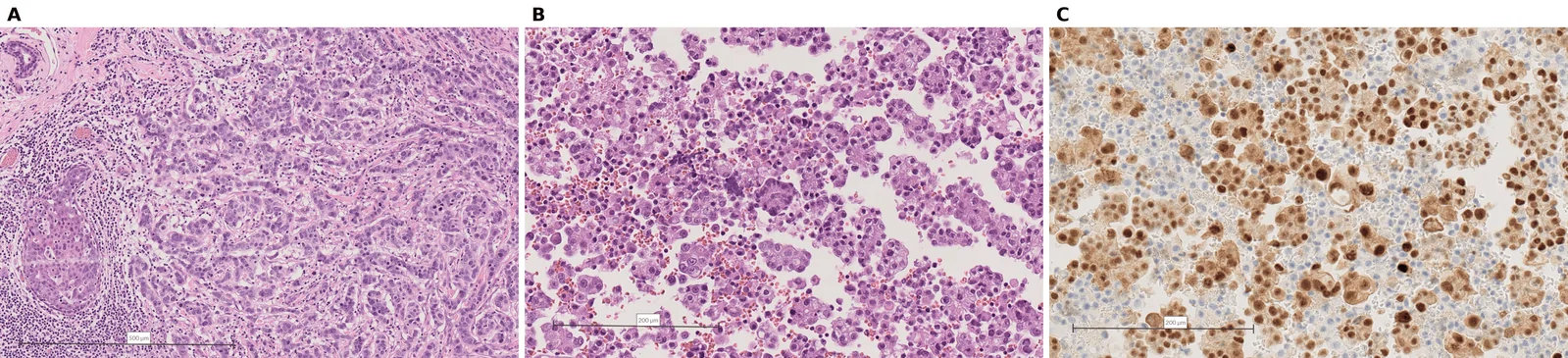
Histopathological and immunohistochemical findings in the daughter and mother (A) H&E-stained section of the daughter’s breast tumor showing invasive ductal carcinoma (scale bar, 500 μm). (B) H&E-stained cell block preparation from the mother’s ascitic fluid showing clusters of adenocarcinoma cells (scale bar, 200 μm). (C) PAX8 immunohistochemistry of the mother’s ascitic fluid cell block showing nuclear positivity in tumor cells, supporting Müllerian origin (scale bar, 200 μm). H&E: hematoxylin and eosin; PAX8: paired box 8

## Discussion

This case illustrates several clinically important points. First, BRCA-associated cancer predisposition within a family should not automatically be attributed to a single variant. Prior reports have described BRCA1/BRCA2 double heterozygosity and co-occurring pathogenic variants [5-7]. Unlike classic double heterozygosity within a single individual, the present family demonstrates discordant familial segregation of independent BRCA1 and BRCA2 pathogenic variants across different relatives. The key clinical lesson is that cascade testing restricted to a single known familial variant may result in incomplete hereditary cancer risk assessment. Thus, the significance of this case lies not simply in the coexistence of two BRCA pathogenic variants, but in the counseling challenge created by their discordant segregation within the family.

Second, this case highlights the potential under-recognition of paternal inheritance. BRCA1/2 pathogenic variants are inherited in an autosomal dominant manner, and either maternal or paternal lineages may transmit risk [1]. However, male carriers may remain unaffected or may not develop cancers that prompt genetic evaluation. In the present family, the father was deceased and untested, and there was no significant paternal cancer history. Because his age at death and detailed age information for paternal relatives were unavailable, the apparently negative paternal family history should be interpreted cautiously. The absence of a suggestive paternal cancer history did not exclude possible paternal transmission of BRCA2; however, because the father was untested, a de novo origin also remains possible. Further segregation testing in paternal relatives, if feasible, could help clarify the origin of the variant.

Third, the case demonstrates the importance of distinguishing tumor testing from germline testing. myChoice CDx is a tumor-based companion diagnostic assay that evaluates BRCA1/2 alterations and homologous recombination deficiency-related genomic instability in ovarian cancer tissue [9]. Detection of a BRCA alteration in tumor tissue can raise suspicion for HBOC but requires germline testing when hereditary risk and cascade testing are being considered. In the mother, germline confirmation was essential to establish HBOC and to interpret risk for her children and other relatives.

Fourth, this case underscores both the utility and limitations of targeted cascade testing. Testing for a known familial pathogenic variant is an efficient and cost-effective approach for identifying relatives who have inherited that variant [8]. However, by design, it does not detect pathogenic variants outside the targeted region or in other cancer predisposition genes. Thus, a negative result for a known familial variant should not be considered to exclude hereditary cancer susceptibility when an individual’s personal or family history remains discordant with that variant. In this family, the daughter underwent comprehensive germline BRCA1/2 testing before the mother’s tumor-detected BRCA1 deletion was confirmed in the germline. Had subsequent cascade testing been restricted solely to the maternal BRCA1 deletion, additional familial BRCA variants such as the daughter’s BRCA2 variant could potentially have been missed. Consequently, families harboring more than one pathogenic variant may remain underrecognized when cascade testing is restricted to a single known familial variant [7,8]. In Japan, national health insurance coverage for germline BRCA1/2 testing was expanded in June 2026 to parents, children, and siblings of individuals diagnosed with HBOC by germline BRCA1/2 testing when the relative is suspected of having HBOC [10]. This policy change is expected to increase access to cascade testing among at-risk relatives and highlights the importance of genetic counseling to determine whether testing should be limited to a known familial variant or broadened according to the individual’s personal and family history.

Finally, the case has practical implications for genetic counseling. The daughter’s children each have a 50% risk of inheriting her confirmed BRCA2 pathogenic variant. In contrast, the daughter’s siblings have a 50% risk of inheriting the maternal BRCA1 deletion, but their risk of carrying the daughter’s BRCA2 variant is uncertain because the father was not tested and the variant could have arisen de novo in the daughter. Accordingly, BRCA2 testing in the siblings should be considered on an individualized basis, taking into account the unresolved origin of the variant and the limited information available from the paternal family history.

## Conclusions

Different BRCA pathogenic variants may coexist within a single pedigree, even when one parental lineage lacks an obvious BRCA-associated cancer history. This case emphasizes that clinicians should avoid assuming that a known familial BRCA variant explains all hereditary risk in the family. Comprehensive germline evaluation and individualized cascade testing strategies should be considered when clinical or familial findings are discordant to reduce the risk of incomplete assessment.
